# The Pterygoid Anchorage Spatial Adequacy Concept: A CT-Based Morphometric Framework for Pterygoid Implant Planning

**DOI:** 10.3390/jcm15218388

**Published:** 2026-10-28

**Authors:** Bartosz Bielecki-Kowalski, Matteo Giorgi, Natalia Bielecka-Kowalska, Daria Adrianowicz, Katarzyna Bogusiak

**Affiliations:** 1 Department of Maxillofacial Surgery, Medical University of Lodz, 251 Pomorska St., 92-213 Lodz, Poland; 2 Department of Dentistry, Vita-Salute University, IRCCS San Raffaele Hospital, 20132 Milan, Italy; 3 Department of Oral Mucosal and Periodontal Diseases, Medical University of Lodz, 251 Pomorska St., 92-213 Lodz, Poland; 4 Student Scientific Circle of Maxillofacial Surgery, Medical University of Lodz, 251 Pomorska St., 92-213 Lodz, Poland; daria.adrianowicz@stud.umed.lodz.pl

**Keywords:** dental implants, greater palatine canal, implant planning, morphometric analysis, pterygoid implants, tomography, X-ray computed

## Abstract

**Background/Objectives:** Pterygoid implant placement remains technically demanding due to the complex three-dimensional anatomy of the pterygomaxillary region and its close relationship with critical neurovascular structures. The aim of this study was to introduce the Pterygoid Anchorage Spatial Adequacy (PASA) concept, a computed tomography (CT)-based morphometric framework for evaluating anatomical conditions relevant to pterygoid implant placement. **Methods:** A total of 157 CT scans were included, providing 314 pterygo-maxillo-palatal regions for analysis. Measurements included the distance between the pterygomaxillary fissure and greater palatine canal (PF–GPC), the maxillary tuberosity and greater palatine canal (MT–GPC), pterygoid process height (PPH), and anterior corridor width (PWW-1). **Results:** PF–GPC ≤ 4 mm was found in 69 regions (22.0%) and was associated with greater PPH and lower MT–GPC values. Age showed negative associations with MT–GPC and PWW-1, while the presence of posterior maxillary molars was associated with wider anterior corridor dimensions and larger angular parameter values. The PF–GPC distance demonstrated substantial interindividual variability, indicating that neurovascular clearance cannot be assumed to be constant. **Conclusions:** The PASA concept integrates neurovascular clearance, pterygoid morphology, maxillary dimensions, and dentition-related changes into a structured anatomical assessment and may contribute to more individualized preoperative anatomical assessment of pterygoid implant placement.

## 1. Introduction

Pterygoid implants have become a key component of posterior maxillary rehabilitation, particularly in cases of severe maxillary atrophy, pneumatized sinuses, or compromised posterior dentition [1,2,3]. Their ability to engage the dense cortical bone of the pterygoid process of the sphenoid bone provides a biomechanically stable anchorage that often eliminates the need for sinus augmentation or extensive grafting procedures [1,2,3,4,5,6]. Since their introduction by Tulasne, they have gained increasing attention in oral and maxillofacial surgery [1]. Clinical studies and systematic reviews have demonstrated high survival rates and long-term predictability [1,2,3,4,5,6,7].

Despite these advantages, pterygoid implant placement remains technically demanding, largely due to the complex three-dimensional anatomy of the pterygomaxillary region [1,6,8]. The procedure is characterized by limited surgical access, restricted visualization, and close proximity to critical anatomical neurovascular structures [1,6,8]. The trajectory of the implant traverses the maxillary tuberosity, pyramidal process of the palatine bone, and pterygoid process of the sphenoid bone, while neighboring the greater palatine canal and branches of the maxillary artery [1,6,8].

**Figure 1:**
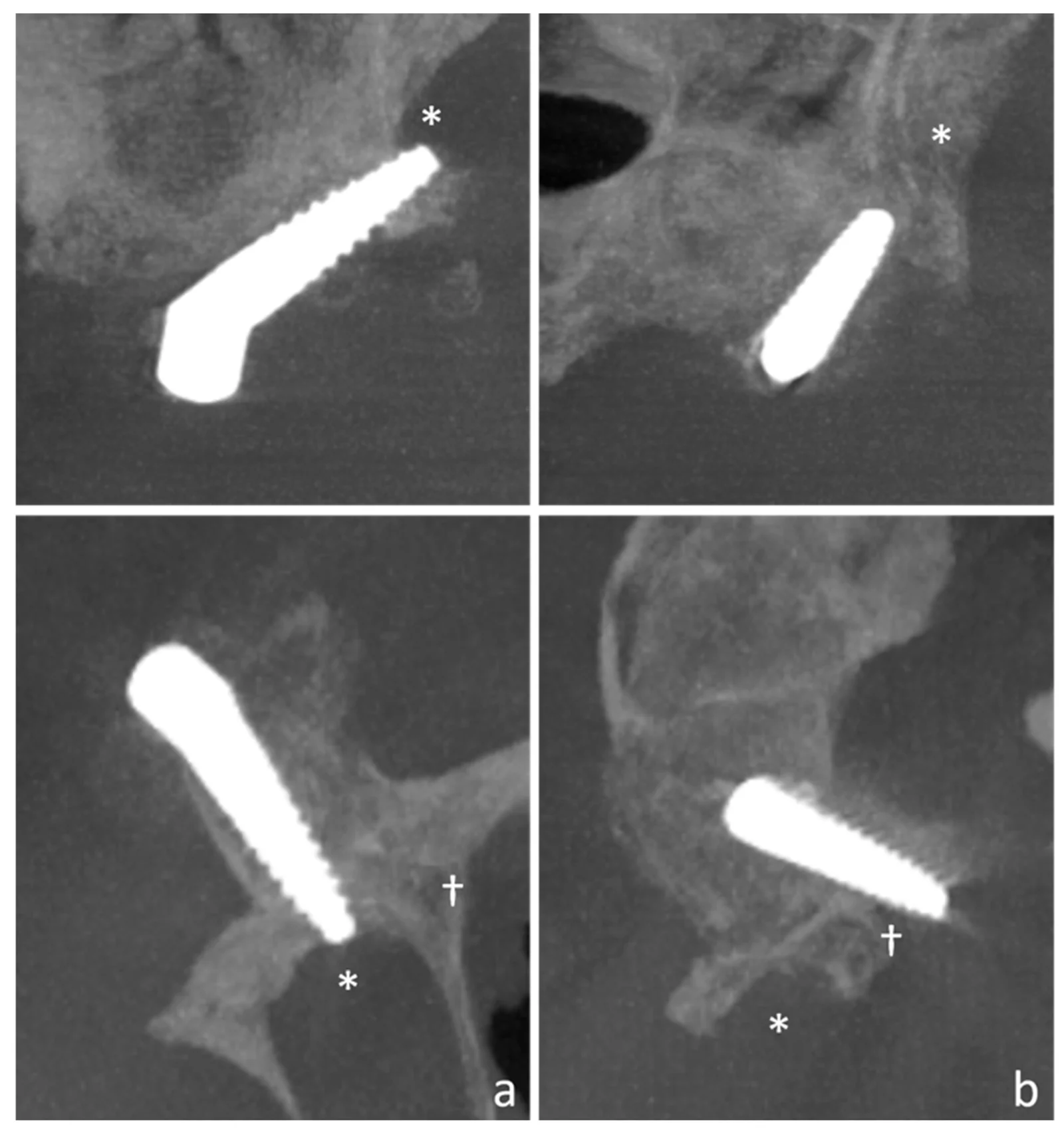
Clinical examples of pterygoid implant positioning. (**a**) Correctly positioned pterygoid implant achieving bicortical stabilization within the pterygoid fossa. (**b**) Incorrect medial implant trajectory, resulting in interference with the greater palatine canal and failure to engage the pterygoid fossa. * pterygoid fossa; † greater palatine canal (GPC).

The clinical relevance of these spatial relationships is illustrated in Figure 1. 

A correctly positioned pterygoid implant follows a trajectory through the maxillary tuberosity and pyramidal process toward cortical engagement within the pterygoid region while maintaining an adequate distance from the greater palatine canal [2,8]. In contrast, excessive buccal inclination may result in insufficient engagement of the intended anchorage zone, whereas excessive medial deviation may lead to interference with the greater palatine canal and its neurovascular contents, as illustrated in Figure 1.

Consequently, successful treatment depends heavily on an accurate preoperative assessment of patient-specific anatomy and precise determination of a safe implant corridor [1,7,8]. To minimize deviations from the planned position and protect vital structures, static computer-assisted implant surgery (SCAIS) is increasingly utilized, where parameter settings such as guided sleeve height and drilling distance below the sleeve significantly influence positioning accuracy [9].

Although several anatomical studies have described the pterygoid process and its surrounding structure, the available literature remains heterogeneous in methodology, imaging modalities, and measurement protocols. Moreover, most existing studies focused on isolated linear dimensions rather than the spatial relationships that define the actual implant corridor [1,2,3]. The lack of a unified morphometric framework limits the ability to objectively evaluate anatomical feasibility in individual patients and anticipate structural constraints. Consequently, preoperative evaluation is often still reliant on subjective interpretations of multiple independent measurements rather than a structured anatomical model.

In particular, the spatial relationship between the pterygomaxillary fissure (PF) and the greater palatine canal (GPC)—an area crucial for avoiding neurovascular injury—has not been systematically quantified in a large patient cohort via computed tomography [1]. As demonstrated in cadaveric investigations, the greater palatine canal represents a critical landmark, highlighting the need to define reliable radiological clearance parameters [1,8]. Furthermore, the morphology of the posterior maxilla is subject to significant remodeling influenced by age-related changes and the presence or absence of posterior teeth [1,8]. These factors may modify the tuberosity width, the distance from the maxillary tuberosity to the greater palatine canal (MT–GPC), and the overall geometry of the pterygoid trajectory [1,8]. To date, their combined impact on the spatial adequacy of the pterygoid corridor has not been comprehensively evaluated [1].

Existing classifications, such as the vascular risk thresholds proposed by Uchida et al., provide clinically meaningful cutoffs (<2 mm, 2–4 mm, >4 mm), but have not been validated in relation to broader morphometric determinants such as pterygoid process height (PPH) or anterior corridor width (PWW-1) [1,8]. The aim of this retrospective imaging study was to propose the Pterygoid Anchorage Spatial Adequacy (PASA) concept as a framework for structured anatomical assessment during pterygoid implant planning. Specifically, we sought to characterize PF–GPC and MT–GPC distances, pterygoid process height, and anterior corridor width; examine their associations with age, sex, and posterior maxillary dentition status; and explore anatomical differences between regions with PF–GPC ≤ 4 mm and >4 mm. The study evaluated radiological anatomy and did not assess implant placement accuracy or clinical outcomes.

## 2. Materials and Methods

The retrospective study was conducted in accordance with the opinion of the Bioethics Committee of the Medical University of Łódź stating that ethical approval was not required (opinion No. RNN/209/23/KE, 11 July 2023).

Inclusion criteria for the study included CT scans of the head performed on patients treated at the Department of Maxillofacial Surgery at the Medical University of Łódź.

**Figure 2:**
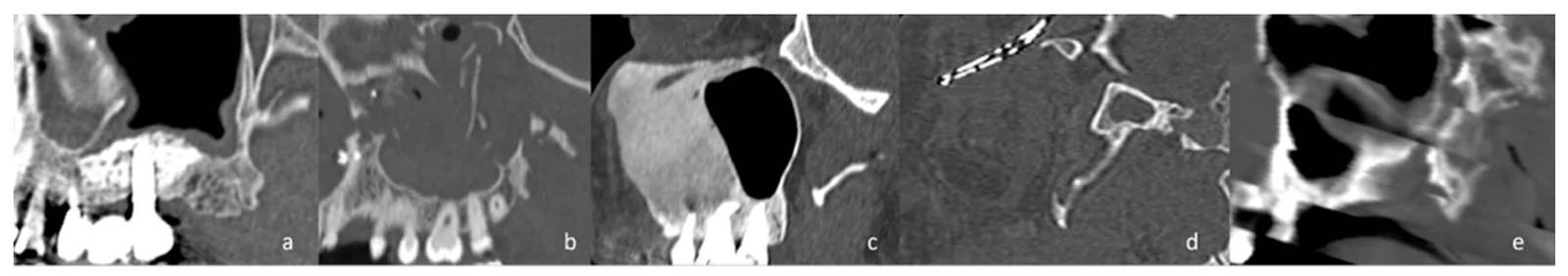
Examples of CT scans excluded from the study: CT scan of a patient following implant reconstruction and sinus augmentation (**a**), CT scan of a post-traumatic patient with a LeFort 1 fracture (**b**), CT scan of a patient with fibrous dysplasia of the maxilla (**c**), CT scan of a patient following resection of the maxilla and orbital floor due to squamous cell carcinoma of the maxillary sinus (**d**), and CT scan containing numerous motion artifacts (**e**).

Exclusion criteria included abnormalities of the anatomical structures of the analyzed areas of the sphenoid bone, maxilla, and palatine bone resulting from trauma, proliferative diseases, inflammatory conditions, or congenital or acquired deformities. We also excluded CT scans of patients who had undergone surgery in the area of the maxillary, sphenoid, and palatine bones, as well as CT scans of poor quality and those containing artifacts that affected the clarity of the examined area (Figure 2). 

A total of 157 computed tomography scans of Caucasian patients met the inclusion and exclusion criteria: 28 cone-beam CT (CBCT) scans (Carestream CS 9300 3D, Carestream Dental LLC, Atlanta, GA, USA) and 129 fan-beam CT (FBCT) scans (Aquilion ONE, Toshiba, Otawara, Japan). The data underwent an anonymization process. From the resulting CT database, 314 regions of the sphenoid, palatine, and maxillary bones were extracted, and anatomical measurements were then applied to them. For each pterygo-maxillo-palatal region, the following structures were identified: processus pterygoideus, pterygomaxillary fissure (PF), maxillary tuberosity (MT), hamular notch (HN) and the greater palatine canal (GPC). In addition, the presence of 1, 2, and 3 upper molars was determined.

**Figure 3:**
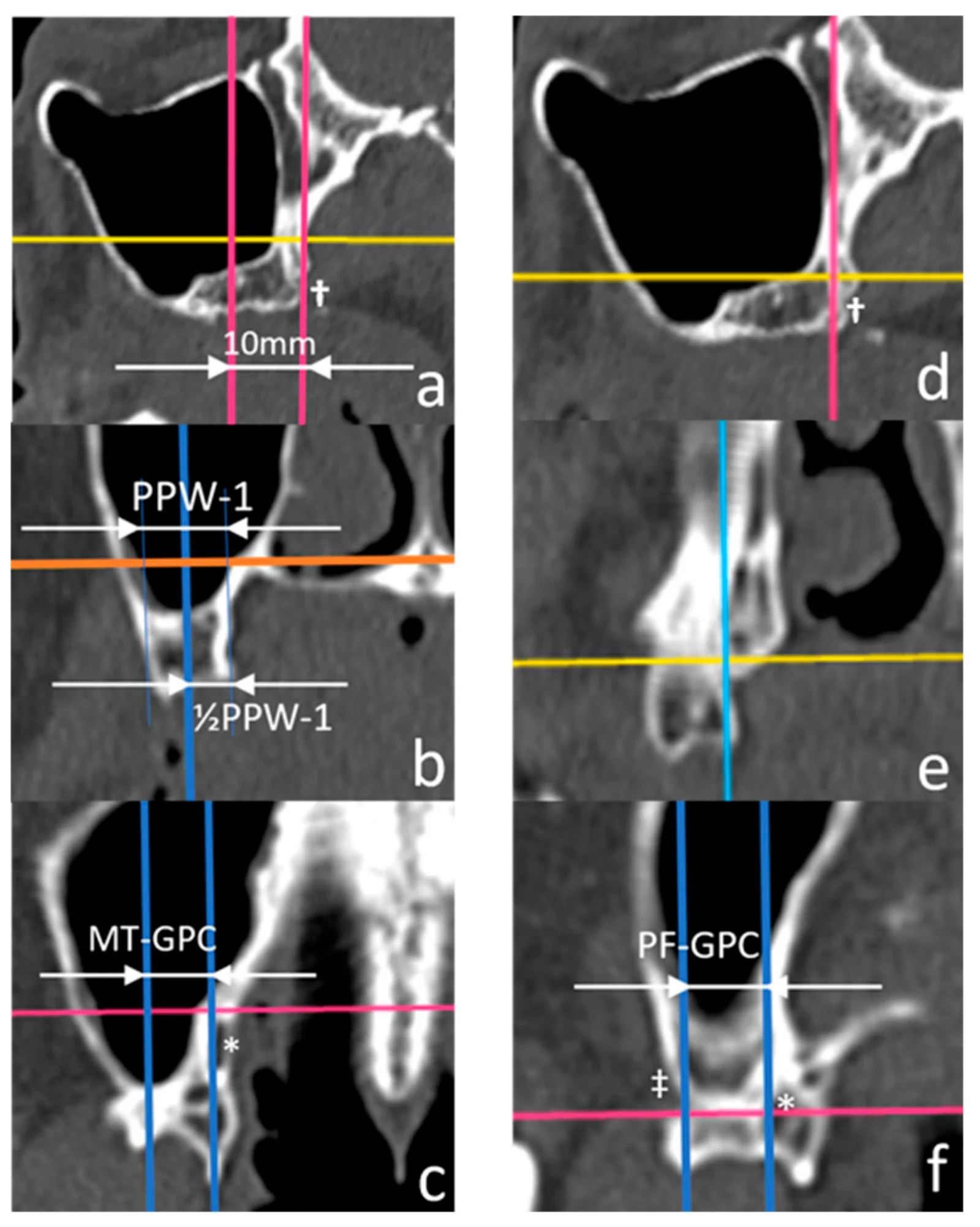
Methods for obtaining linear measurements: MT–GPC (**a**–**c**) and PF–GPC (**d**–**f**). PWW-1—width of the maxillary alveolar process at a distance of 10 mm from HN; * GPC, † HN, ‡ PF.

All CT scans were aligned in the Frankfurt plane, and then linear measurements of PF–GPC, representing the shortest distance between the planes passing through the most mesial point of the PF and the most distal point of the GPC in the axial view, were performed; the MT–GPC measurement, representing the shortest distance between the sagittal planes passing through the most distally located point of the GPC in the axial view and the MT point, located in the middle of the alveolar process of the maxilla, measured 10 mm anterior to the HN (Figure 3). 

**Figure 4:**
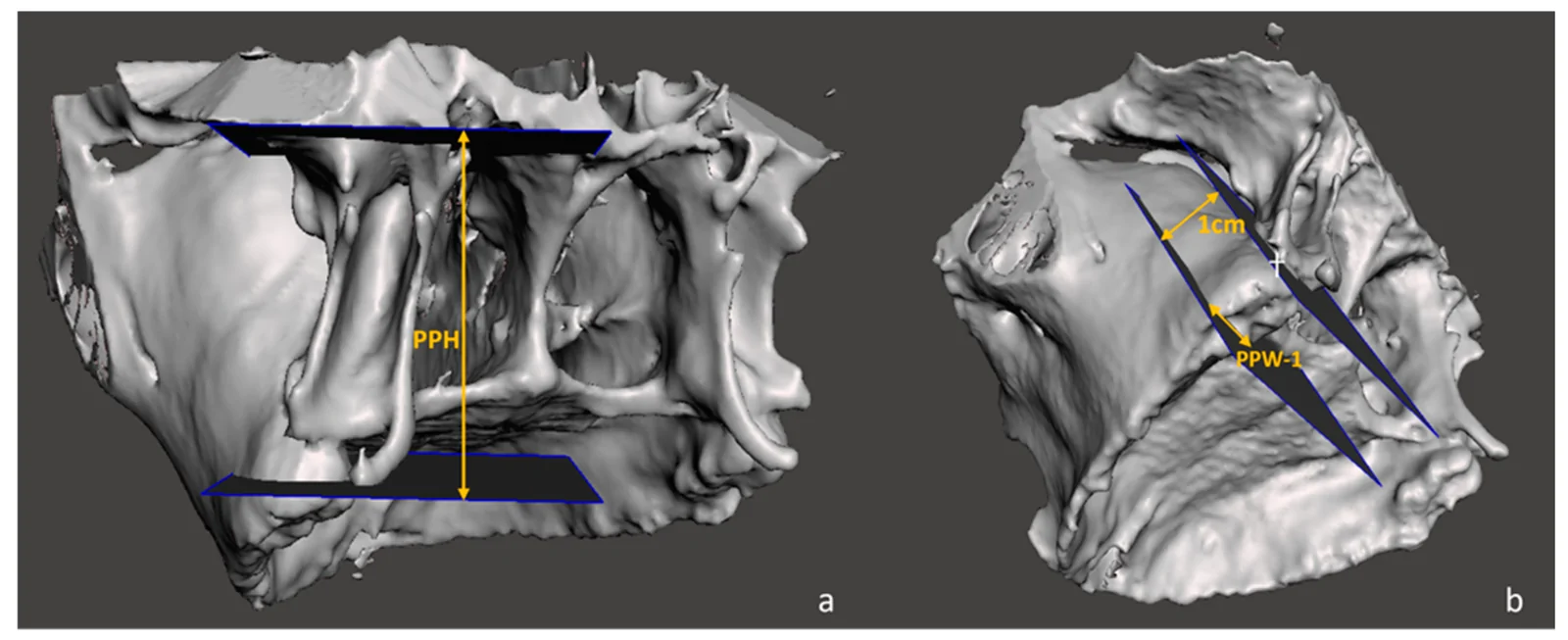
Methods for obtaining measurements of the pterygoid process height (PPH) (**a**) and the width of the alveolar process of the maxilla 1 cm prior to the pterygoid process (PWW-1) (**b**). † HN.

The measurements were performed using the RadiAnt DICOM Viewer software (Medixant, Poznań, Poland; www.radiantviewer.com, accessed on 9 April 2024). Further stages of the study were performed on stereolithographic STL models obtained through segmentation carried out using the software RadiAnt Digital Imaging and Communications in Medicine DICOM Viewer (Medixant, Poznań, Poland; www.radiantviewer.com, accessed on 9 April 2024, ver. 2024.1) and Meshmixer (Autodesk, San Rafael, CA, USA; www.autodesk.com, accessed on 9 April 2024, ver. 3.5.474). The measurements were applied on the created models. Pterygoid process height (PPH), defined as the perpendicular distance between two parallel reference planes: the superior plane passing through the cranial base at the level of the superior attachment/root of the pterygoid process, and the inferior plane passing through the lowest point of the pterygoid hamulus. Pomiar width 1 cm prior to pterygoid proces (PPW-1) measured from the hamular notch. PF–GPC and MT–GPC were measured on axial CT images, whereas PPH and PWW-1 were measured on segmented three-dimensional models. These procedures assessed distinct anatomical dimensions and were not used interchangeably to measure the same parameter (Figure 4).

For each model, the angular geometric coefficient describing the pterygoid corridor was calculated (αMT–GPC):

$$\alpha MT-GPC=\arctan\left(\frac{MT-GPC}{10}\right)$$

We have also revised the terminology in Section 2 by replacing expressions suggesting clinically established vascular risk or acceptable safety margins with descriptive radiological distance categories.

All measurements were performed by a single investigator using the same anatomical landmark definitions, reference planes, and measurement procedures across all examinations.

The measurements obtained and the calculated coefficients and angles were subjected to statistical analysis using Jamovi software (version 2.6.44, dated 13 January 2026). Sensitivity analysis restricted to adults aged ≥18 years compared correlation coefficients and group medians with those in the sample with recorded age. The association analyses were exploratory. Reported *p*-values were not adjusted for multiple comparisons

## 3. Results

In our database, the age and gender distribution was as follows: 214 records were obtained from CT scans of men, and 100 from women. Age distribution: 41.42 ± 18.09 years; median 37.00 [26.00–54.00]; range 8–82. A total of 56 models were obtained through CBCT segmentation, and 258 through FBCT segmentation.

The distribution of anatomical measurements, calculated coefficients, and the presence of 1 molar, 2 molars, and a wisdom tooth are shown in the tables below (Table 1 and Table 2).

**Figure 5:**
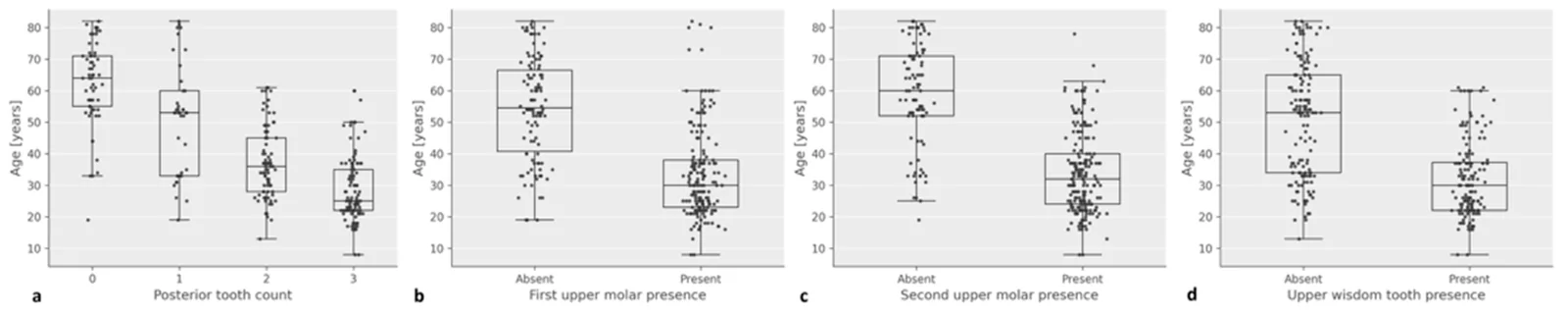
Associations between age and posterior dentition status. Boxplots showing age distributions according to posterior tooth count (**a**), presence of the first upper molar (**b**), second upper molar (**c**), and upper wisdom tooth (**d**).

The measurement results and coefficients were not significantly different depending on the type of computed tomography. Age was significantly correlated with posterior tooth count (rho = −0.693; *p* < 0.001) (Figure 5).

**Figure 6:**
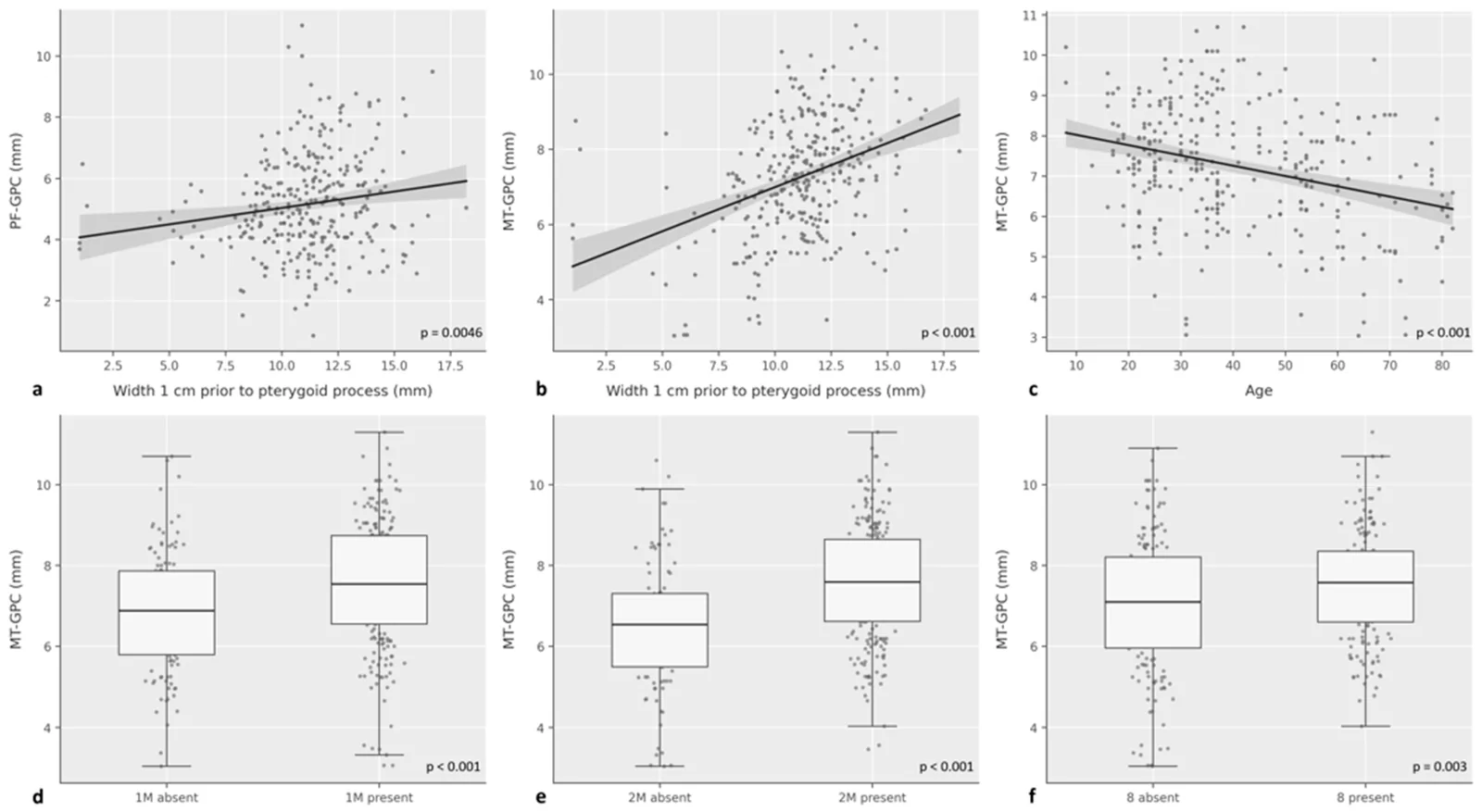
GPC-related morphometric relationships within the pterygoid implant corridor, PF–GPC distance and anterior pterygoid corridor width measured 1 cm anterior to the pterygoid process (**a**), relationship between MT–GPC distance and anterior PWW-1 (**b**), and relationship between MT–GPC distance and age. Increasing age was associated with a lower MT–GPC distance (**c**). MT–GPC distance according to the presence of the first upper molar. Cases with the first molar present showed higher MT–GPC values than cases without the first molar (**d**). MT–GPC distance according to the presence of the second upper molar. The second molar showed the strongest association with MT–GPC distance (**e**). MT–GPC distance according to the presence of the upper wisdom tooth. Cases with the upper wisdom tooth present showed higher MT–GPC values than cases without it (**f**).

Subsequent stages of the statistical analysis involved assessing the relationships between “danger zones” in the context of pterygoid implantology—defined as the distance from the GPD, the critical zone for the occurrence of potential neurovascular complications. The relationships between MT–GPC and PF–GPC were examined, as well as their associations with age, sex, the presence of maxillary molars, and the width of the maxillary alveolar process measured 1 cm from the HN (PWW-1), as well as the height of the pterygoid process (PPH). The 1-cm distance is not accidental; it represents a typical “entry point” for pterygoid implants, as well as the site for mounting the multiunit and the prosthetic superstructure. MT–GPC showed statistically significant correlations with PWW-1 (*p* < 0.001), age (*p* < 0.001), and the presence of 1 (*p* < 0.001), 2 (*p* < 0.001), and 3 upper molars (*p* = 0.003). PF–GPC correlated strongly statistically only with PWW-1 (*p* = 0.046) (Figure 6, Table 3).

Subsequent stages of the analysis involved assessing our group for neurovascular risk according to the classification by Uchida et al. Initially, a predictive model was created using PF–GPC < 2 mm as the basis for a binary variable. The analysis revealed PF–GPC < 2 mm for 4 records (1.3%) and PF–GPC ≥ 2 mm for 310 records (98.7%). A further analysis was conducted by adding an intermediate threshold value of PF–GPC [2–4 mm]. Results of the two threshold analyses: PF–GPC < 2 mm—4 records (1.3%), PF–GPC [2–4 mm]—65 records (20.4%), and PF–GPC > 4 mm—245 records (78.3%). For further comparative analysis, the PF–GPC < 2 mm and PF–GPC 2–4 mm groups were combined into a single exploratory radiological category, PF–GPC ≤ 4 mm, comprising 69 records (22.0%). This grouping allowed for a more stable comparison with the PF–GPC > 4 mm group without implying a clinically validated safety threshold.

**Figure 7:**
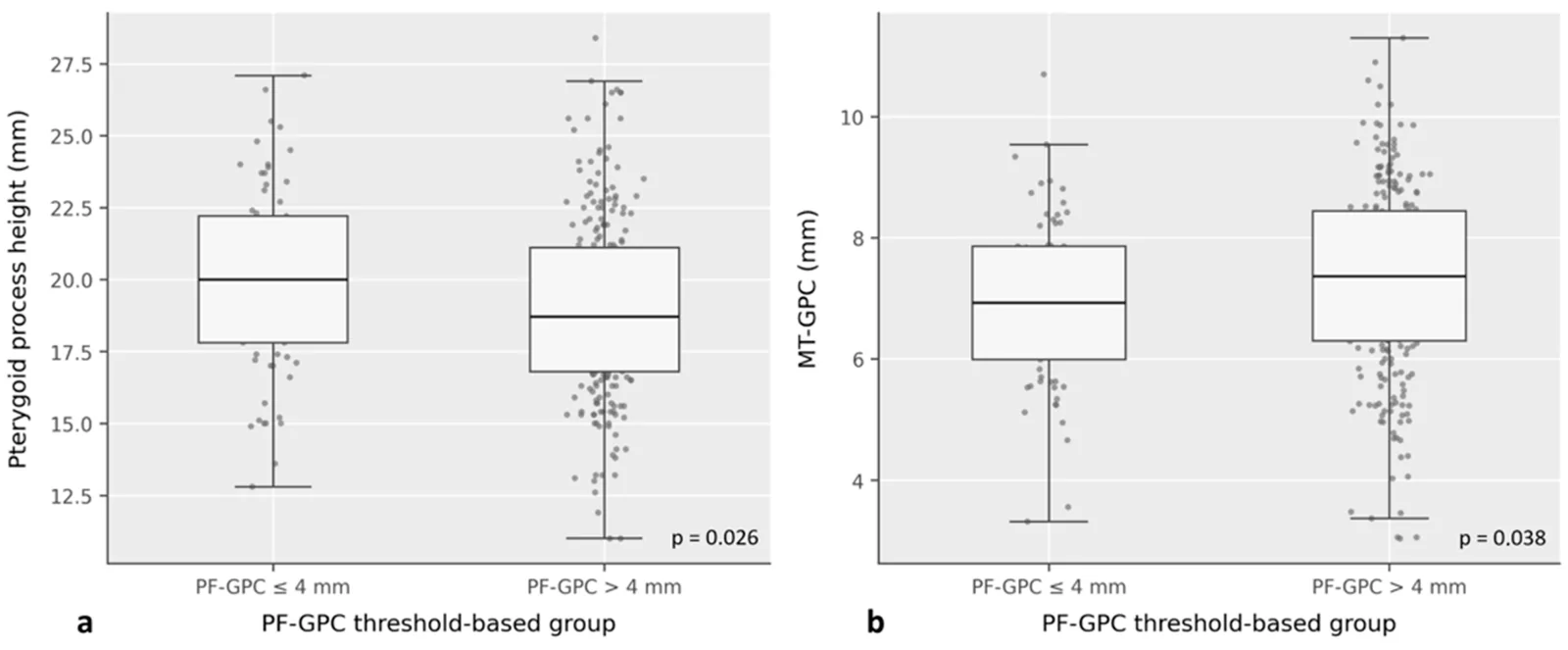
(**a**) PPH vs. PF–GPC threshold-based clearance groups. The PF–GPC ≤ 4 mm group represents limited GPC-related clearance, whereas PF–GPC > 4 mm represents wider clearance. Boxplots are shown with overlaid raw data points. The limited-clearance group demonstrated significantly greater pterygoid process height than the wider-clearance group (median 20.0 mm vs. 18.7 mm; Mann–Whitney U test, *p* = 0.026). (**b**) MT–GPC distance according to PF–GPC threshold-based clearance groups. The PF–GPC ≤ 4 mm group represents limited GPC-related clearance, whereas PF–GPC > 4 mm represents wider clearance. Boxplots are shown with overlaid raw data points. MT–GPC was significantly lower in the PF–GPC ≤ 4 mm group than in the PF–GPC > 4 mm group (median 6.93 mm vs. 7.36 mm; Mann–Whitney U test, *p* = 0.038).

The relationships between the binary division of the group into PF–GPC ≤ 4 mm and PF–GPC > 4 mm and age, PWW-1, MT–GPC, the presence of molars, and the height of the pterygoid process were analyzed. PWW-1 values and the presence of molars did not correlate with the classification. A statistically significant relationship was found between PPH and MT–GPC and the binary PF–GPC classification relative to the 4-mm cutoff value (Figure 7, Table 4).

**Figure 8:**
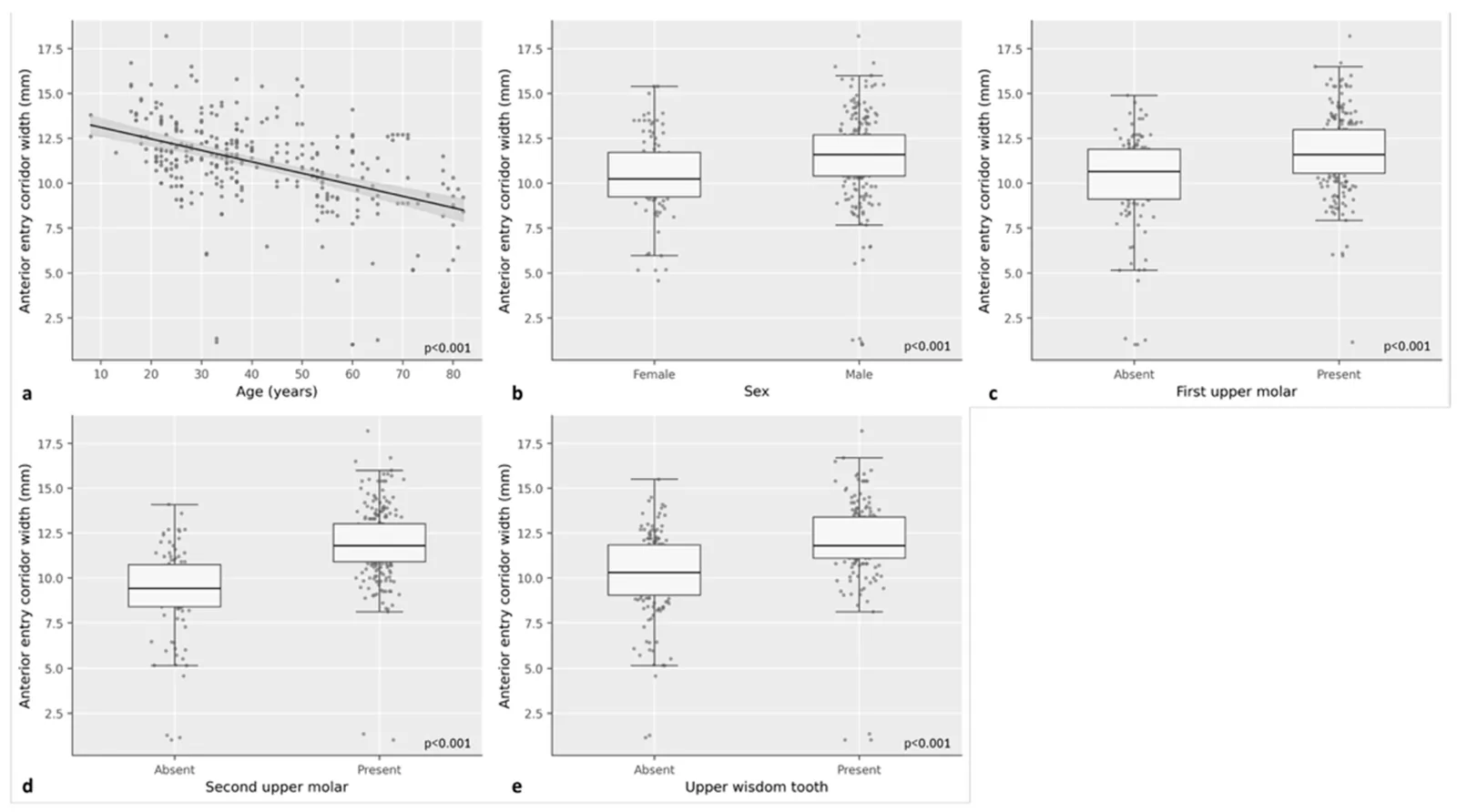
Scatterplot showing the negative association between PWW-1 and age (**a**), boxplot showing greater PWW-1 values in males than in females (**b**), boxplots showing greater PWW-1 values in cases with the first upper molar present (**c**), the second upper molar present (**d**), and the upper wisdom tooth present (**e**). Panels are presented as boxplots with overlaid raw data points or scatterplots with linear fit and 95% confidence band.

In the next stage, the entry point for a typical pterygoid implant was examined. Correlations were determined between PWW-1 and age, sex, and the presence of 1M, 2M, and 3M. Statistically significant results were obtained for the relationships between PWW-1 and age (rho = −0.469, *p* < 0.001), sex (*p* < 0.001), the presence of the first upper molar (*p* < 0.001), the presence of the second upper molar (*p* < 0.001), and the presence of the upper wisdom tooth (*p* < 0.001) (Figure 8, Table 5).

The next part of the statistical analysis involved the radiological angular-trajectory parameter (αMT–GPC) associated with MT–GPC. The angular parameter αMT–GPC, calculated as arctan(MT–GPC/10 mm), was available in 314 cases. The mean value of αMT–GPC was 35.39 ± 5.93°, and the median was 36.02° [IQR: 31.73–39.74°], with a range from 16.91° to 48.49°.

αMT–GPC showed a significant negative correlation with age (rho = −0.267, *p* < 0.001) and a positive correlation with the width of the anterior pterygoid corridor entry-point PWW-1 (rho = 0.364, *p* < 0.001). A weak but statistically significant positive correlation was also found between αMT–GPC and PF–GPC (rho = 0.207, *p* < 0.001).

**Figure 9:**
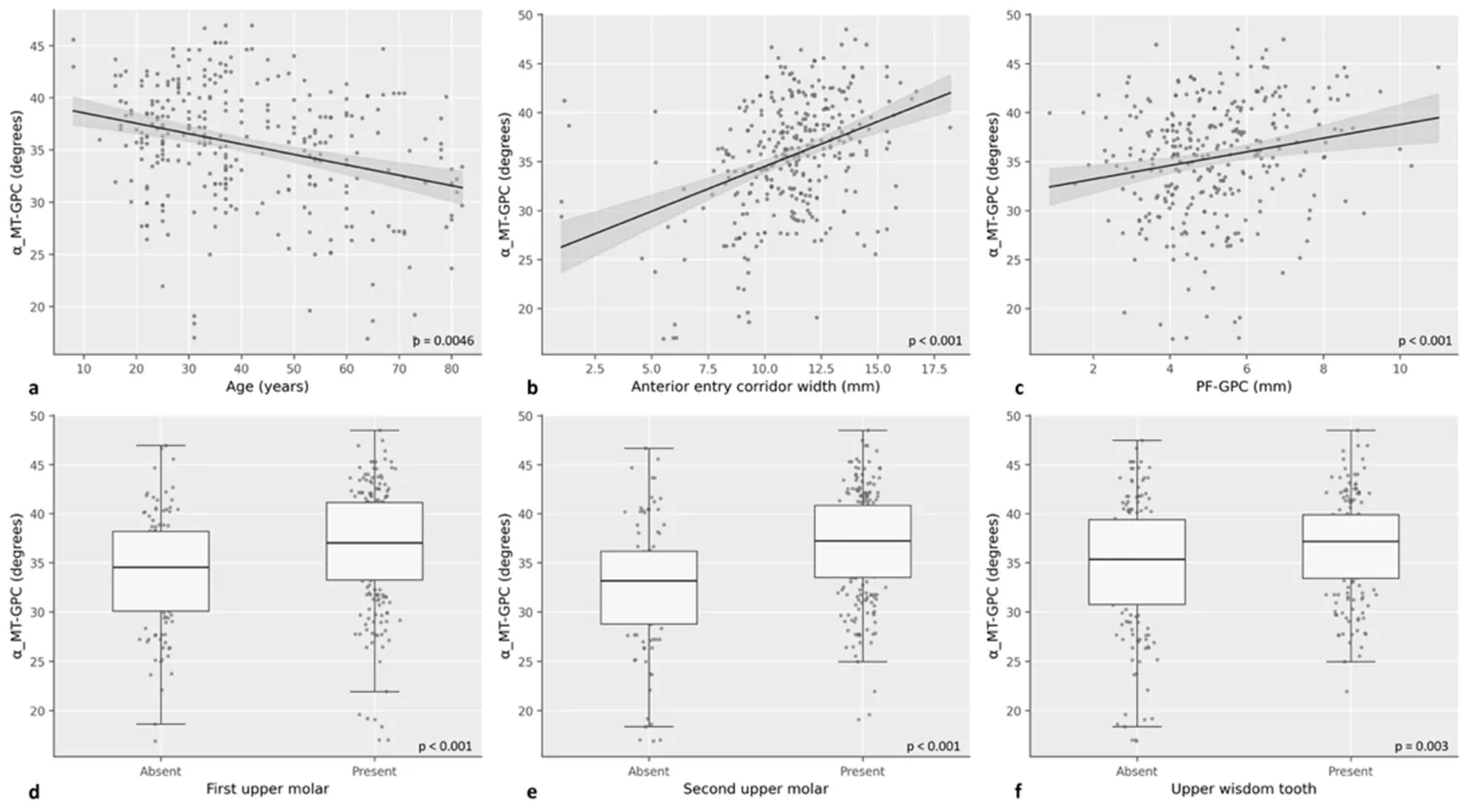
Significant relationships of the angular parameter α_MT–GPC. scatterplot showing the negative association between α_MT–GPC and age (**a**), scatterplot showing the positive association between α_MT–GPC and PWW-1 (**b**), scatterplot showing the positive association between α_MT–GPC and PF–GPC (**c**), boxplots showing greater α_MT–GPC values in cases with the first upper molar present (**d**), the second upper molar present (**e**), and the upper wisdom tooth present (**f**). Panels are presented as boxplots with overlaid raw data points or scatterplots with linear fit and 95% confidence band.

No significant differences in αMT–GPC were found between sex, pterygoid process height, and PF–GPC threshold-based groups. However, the presence of maxillary molars was associated with higher αMT–GPC values. These differences were significant for the first maxillary molar (median 37.02° vs. 34.53°, *p* < 0.001), the second maxillary molar (37.22° vs. 33.16°, *p* < 0.001), and the maxillary wisdom tooth (37.16° vs. 35.36°, *p* = 0.003) (Figure 9, Table 6).

In the adult-only sensitivity analysis, the examined associations retained their direction and showed similar magnitudes. Correlations of age with MT–GPC and PWW-1 were slightly weaker (Spearman’s rho: −0.249 and −0.431, respectively). Regions with PF–GPC ≤ 4 mm continued to show higher median PPH and lower median MT–GPC than regions with PF–GPC > 4 mm.

## 4. Discussion

The present study introduces the Pterygoid Anchorage Spatial Adequacy (PASA) concept as a CT-based morphometric framework for assessing anatomical conditions relevant to pterygoid implant planning. Rather than focusing on isolated linear measurements, the proposed approach integrates spatial relationships between the maxillary tuberosity, pterygomaxillary fissure, greater palatine canal, and pterygoid process morphology. The principal findings were threefold. First, the PF–GPC distance demonstrated substantial interindividual variability, confirming that the neurovascular safety margin in the pterygomaxillary region cannot be assumed to be constant [2,8].

Second, reduced PF–GPC clearance was associated with greater pterygoid process height (PPH) and lower MT–GPC values, indicating that anatomical constraints are interconnected rather than independent. Third, age-related posterior maxillary remodelling and loss of posterior dentition were associated with narrowing of the anterior pterygoid corridor and reduction in several parameters relevant to implant planning [2,7].

One of the most clinically relevant findings concerns the distribution of PF–GPC values. Uchida et al. reported a mean PF–GPC distance of 2.9 mm and a minimum value of 0.2 mm in a cadaveric Japanese population, while 26 out of 78 hemi-heads demonstrated PF–GPC distances below 2 mm, highlighting substantial anatomical variability within the pterygomaxillary region. The authors emphasized that implant planning should therefore be based on careful individual radiological assessment rather than on average anatomical values [8]. Our findings are consistent with these observations. However, we additionally found that the inclusion of cases with PF–GPC values between 2 and 4 mm in the limited-clearance category revealed significant associations with pterygoid process height and MT–GPC distance, suggesting that anatomical constraints are not confined exclusively to the most extreme PF–GPC values. On this basis, we considered PF–GPC ≤ 4 mm as an exploratory radiological grouping to examine anatomical differences beyond the most extreme PF–GPC values described by Uchida et al. [8].

Several factors support this more conservative approach. First, the study by Uchida et al. was performed exclusively on Japanese cadaveric specimens [8]. This is important because craniofacial dimensions and cephalometric norms differ between populations, including Japanese and Caucasian cohorts [10,11]. The current study evaluated a Caucasian population, in whom larger maxillary dimensions may potentially require the use of longer pterygoid implants to achieve engagement of the pterygoid process and obtain adequate cortical anchorage. Rodríguez et al. reported that implants of at least 15 mm are generally required to fully exploit the available bone stock of the pterygomaxillary region [7]. This is supported by clinical data showing higher osseointegration rates for longer implants in the pterygomaxillary region [12]. Longer implants inherently increase the consequences of even minor angular deviations during drilling and insertion, potentially reducing the effective safety margin around adjacent anatomical structures [9,13].

Second, the pterygomaxillary region presents unique biomechanical and anatomical challenges. Previous anatomical studies have demonstrated substantial heterogeneity of bone density along the implant trajectory [3,7]. The implant pathway traverses the relatively low-density maxillary tuberosity before engaging the dense cortical bone of the pyramidal and pterygoid processes [3,6,7].

Rodríguez et al. demonstrated that bone density in the pterygoid region is markedly higher than within the tuberosity itself [7]. Such abrupt transitions between low-density cancellous bone and dense cortical structures may facilitate drill deflection and trajectory deviation, particularly during deep osteotomy preparation. Furthermore, engagement of a curved cortical surface at the pterygoid process may increase the tendency for drill skidding and redirection. Under these circumstances, a radiological clearance of only 2–4 mm may not provide the same practical safety margin as suggested by static anatomical measurements.

Particular attention should also be paid to the junction between the pyramidal process of the palatine bone and the medial pterygoid plate of the sphenoid bone. From a clinical perspective, this region may constitute an important terminal anchorage zone because engagement of its dense cortical structures may contribute to high primary stability. However, successful access to this area depends on an appropriately selected implant trajectory and must be balanced against the proximity of the greater palatine canal and the descending palatine neurovascular bundle. Thus, the junction should be considered as a potential biomechanical target, whereas PF–GPC remains a direct radiological parameter of neurovascular clearance.

Third, the increasing adoption of static computer-assisted implant surgery does not completely eliminate the risk of positional inaccuracies. El Kholy et al. demonstrated that drilling accuracy deteriorates as the free drilling distance below the guide sleeve increases [9]. This observation is particularly relevant for pterygoid implants, where the osteotomy is often considerably longer than in conventional implant placement and the endpoint of the preparation is located far from the guiding sleeve [9]. Moreover, systematic and clinical evidence indicates that computer-guided implant placement, although accurate, is still associated with clinically relevant deviations that must be considered during treatment planning [13]. Mechanical factors related to the tolerance between surgical guide components may further contribute to implant-positioning error [14,15]. In the pterygoid region, these inaccuracies may be amplified by limited access, reduced visibility, posterior implant location, and the technical sensitivity of the procedure [6,16]. Collectively, these factors support the notion that anatomical clearances should be interpreted in conjunction with expected surgical deviation rather than as purely static measurements.

An additional important finding was the relationship between PF–GPC clearance and pterygoid process morphology. In this sample, regions with PF–GPC ≤4 mm had greater PPH values and lower MT–GPC distances. To our knowledge, this relationship has not been previously reported. Existing studies have largely focused on isolated measurements describing either the pterygoid process or the vascular corridor [2,3,7,8,17]. The present findings suggest that vertical development of the pterygoid process is associated with narrowing of the neurovascular corridor, indicating that complex anatomical interactions contribute to the overall feasibility of pterygoid anchorage. This observation supports the rationale behind the PASA concept, in which anatomical suitability is evaluated as a multidimensional construct rather than through a single measurement.

The influence of age and posterior dentition status also deserves consideration. Increasing age and reduced posterior dentition were associated with lower MT–GPC and PWW-1 values. These findings may be clinically relevant because patients considered for pterygoid implant rehabilitation frequently present with posterior maxillary atrophy and partial or complete edentulism. However, due to the retrospective cross-sectional design, these relationships should be interpreted as associations rather than causal effects.

These findings are consistent with the well-recognized phenomenon of posterior maxillary remodelling following tooth loss [5,7,18]. Progressive alveolar resorption and sinus pneumatization alter the morphology of the maxillary tuberosity and may reduce the available implant corridor [5,7,18]. From a clinical perspective, these results indicate that older edentulous patients may represent a subgroup requiring particularly careful preoperative assessment because anatomical conditions favourable for pterygoid anchorage gradually deteriorate with age and tooth loss.

The derived angular parameter αMT–GPC describes anatomical corridor geometry; it does not represent a validated implant trajectory, as no virtual or clinical implant placement was performed in the analyzed datasets. Greater αMT–GPC values were associated with wider anterior corridors, larger PF–GPC distances, and the preservation of posterior dentition. Although this parameter requires further validation, it may provide a useful supplementary descriptor of pterygoid implant geometry by combining information from multiple anatomical landmarks into a single clinically interpretable metric.

At this stage, PASA is intended as a structured framework for reviewing patient-specific anatomical constraints during implant planning, rather than a validated scoring system or clinical decision algorithm. First, it emphasizes that neurovascular safety should be assessed using a combination of PF–GPC, MT–GPC, PPH, and anterior corridor dimensions rather than a single threshold value. Second, it identifies a substantial subgroup of patients with intermediate anatomical clearance (2–4 mm) who may warrant additional caution during treatment planning. Third, it highlights the importance of considering expected surgical deviations, implant length, and guidance system limitations when interpreting radiological safety margins [9,12,13,14,15]. Consequently, preoperative CBCT analysis should be viewed not merely as a tool for measuring distances, but as a means of evaluating the overall spatial adequacy of the pterygoid corridor. At present, PASA should therefore be regarded as an anatomical planning framework rather than a validated predictor of surgical safety or clinical outcomes.

Previous research supports CT-derived three-dimensional reconstructions for craniofacial morphometry [19]. Here, axial CT images and segmented 3D models assessed distinct anatomical dimensions rather than the same parameter. This supports the methodological rationale, but does not validate our specific measurement procedures.

Several limitations should be acknowledged. Measurements were performed by a single investigator, and intra- and inter-observer reproducibility were not formally assessed. Consequently, measurement variability and its potential influence on the reported associations and threshold-based categorization could not be quantified. The retrospective design and exclusive use of radiological data preclude direct correlation with intraoperative findings or clinical complications. Given the number of comparisons and the use of unadjusted *p*-values, the observed associations should be interpreted cautiously and regarded as hypothesis-generating. The study also did not evaluate actual implant placement accuracy, meaning that the relationship between morphometric parameters and surgical outcomes remains indirect. Additionally, the cohort consisted exclusively of Caucasian patients, limiting generalizability of the morphometric findings. Validation in other populations is therefore required.

Future studies should validate the PASA concept prospectively, investigate its association with guided and freehand implant placement accuracy, and determine whether the incorporation of these morphometric variables improves the prediction of surgical complications and treatment outcomes.

## 5. Conclusions

The pterygomaxillary region showed substantial variation in anatomical dimensions relevant to pterygoid implant planning. The proposed PASA concept provides a framework for the structured assessment of these anatomical relationships. The PF–GPC ≤ 4 mm category should be regarded as an exploratory radiological grouping rather than a validated surgical safety threshold. Further validation is required to determine whether PASA informs clinical decisions or improves implant placement accuracy and patient outcomes.

## 6. Future Directions

Future studies should extend the present morphometric framework by directly characterizing the junction between the pyramidal process of the palatine bone and the medial pterygoid plate of the sphenoid bone. Subsequent analyses should include the dimensions, orientation, and cortical thickness of this region to determine its suitability as a terminal anchorage zone and to clarify its potential relationship with primary implant stability. A more detailed evaluation of the pterygoid process, including the dimensions and spatial orientation of its medial and lateral plates, should also be performed. Furthermore, the depth and configuration of the alveolar recess of the maxillary sinus should be incorporated into future models, as sinus pneumatization may affect the available transmaxillary bone corridor, the implant entry point, and the achievable pterygoid trajectory. Integration of these variables with PF–GPC, MT–GPC, PWW-1, and three-dimensional trajectory analysis may enable the development of a more comprehensive patient-specific planning model for pterygoid implant placement.

## Figures and Tables

**Table 1:** Basic pterygoid corridor morphology.

Parameter	N	Mean ± SD	Median [IQR]	Range
PWW-1	314	11.10 ± 2.43 mm	11.30 [9.84–12.60] mm	1.01–18.20 mm
PF–GPC	314	5.15 ± 1.60 mm	5.02 [4.07–6.11] mm	0.86–11.00 mm
MT–GPC	314	7.21 ± 1.54 mm	7.27 [6.18–8.32] mm	3.04–11.30 mm
PPH	314	19.23 ± 3.15 mm	19.00 [17.10–21.39] mm	11.00–27.10 mm
αMT–GPC	314	35.39 ± 5.93°	36.02 [31.73–39.74]°	16.91–48.49°

**Table 2:** Posterior dentition status in the pterygoid subset.

Dental Variable	Absent/Category n (%)	Present n (%)	Comment
First upper molar (1M)	114 (36.3%)	200 (63.7%)	Side-level records
Second upper molar (2M)	96 (30.6%)	218 (69.4%)	Side-level records
Upper wisdom tooth (8)	155 (49.4%)	159 (50.6%)	Side-level records
Posterior tooth count = 0	63 (20.1%)	—	Number of present posterior teeth: 1M + 2M + 8
Posterior tooth count = 1	44 (14.0%)	—	Number of present posterior teeth: 1M + 2M + 8
Posterior tooth count = 2	88 (28.0%)	—	Number of present posterior teeth: 1M + 2M + 8
Posterior tooth count = 3	119 (37.9%)	—	Number of present posterior teeth: 1M + 2M + 8

**Table 3:** Relationships between MT–GPC, PF–GPC, and measurements of PWW-1 and PPH, as well as the presence of the first, second, and third molars.

Predictor	Test	MT–GPC [mm]	*p* Value	PF–GPC [mm]	*p* Value
PWW-1	Spearman correlation	rho = 0.356	**<0.001**	rho = 0.159	**0.005**
1M status	Mann–Whitney U	Absent: 6.80 [5.76–7.87] Present: 7.53 [6.58–8.52]	**<0.001**	Absent: 5.11 [4.09–5.78] Present: 4.93 [4.07–6.29]	0.563
2M status	Mann–Whitney U	Absent: 6.52 [5.46–7.29] Present: 7.58 [6.62–8.50]	**<0.001**	Absent: 5.14 [4.16–5.81] Present: 4.97 [4.07–6.26]	0.837
3M status	Mann–Whitney U	Absent: 6.98 [5.79–8.04] Present: 7.58 [6.64–8.34]	**<0.001**	Absent: 5.12 [4.09–5.90] Present: 4.90 [4.06–6.25]	0.976
Age	Spearman correlation	rho = −0.284	**<0.001**	rho = −0.075	0.206
PPH	Spearman correlation	rho = 0.006	0.914	rho = 0.003	0.916

**Table 4:** Associations between exploratory PF–GPC radiological groups (≤4 mm and >4 mm) and age, PWW-1, PPH, MT–GPC, and the presence of upper molars.

Predictor	Test	PF–GPC ≤ 4 mm	PF–GPC > 4 mm	*p* Value	Univariable OR for PF–GPC ≤ 4 mm (95% CI)
Age	Mann–Whitney U	n = 65; 37.00 [30.00–52.00]	n = 225; 37.00 [25.00–57.00]	0.669	0.99 (0.98–1.01); *p* = 0.400
PWW-1	Mann–Whitney U	n = 69; 11.20 [9.63–12.20]	n = 245; 11.30 [9.85–12.60]	0.583	0.96 (0.86–1.07); *p* = 0.433
PPH	Mann–Whitney U	n = 69; 20.00 [17.80–22.20]	n = 245; 18.70 [16.80–21.10]	**0.026**	1.11 (1.01–1.20); *p* = 0.023
MT–GPC	Mann–Whitney U	n = 69; 6.93 [5.99–7.86]	n = 245; 7.35 [6.29–8.45]	**0.038**	0.85 (0.72–1.02); *p* = 0.074
1M present	Chi-square	45/69 (65.2%)	155/245 (63.3%)	0.876	1.09 (0.62–1.90); *p* = 0.766
2M present	Chi-square	53/69 (76.8%)	165/245 (67.3%)	0.174	1.61 (0.86–2.98); *p* = 0.134
3M present	Chi-square	38/69 (55.1%)	121/245 (49.4%)	0.485	1.26 (0.73–2.15); *p* = 0.405

Notes. In 24 cases, there is no information about the patient’s age at the time of the examination.

**Table 5:** Association between PWW-1 and demographic/dental predictors in the pterygoid subset PWW-1 values are presented as median [IQR], unless otherwise indicated.

Predictor	Test	Group/Statistic	n	PWW-1 Value	*p* Value
Age	Spearman correlation	Continuous variable	290	rho = −0.476	**<0.001**
Sex	Mann–Whitney U	Female	100	10.25 [9.24–11.72]	**<0.001**
Sex	Mann–Whitney U	Male	214	11.50 [10.40–12.70]	**<0.001**
1M status	Mann–Whitney U	Absent	114	10.45 [9.09–11.90]	**<0.001**
	Mann–Whitney U	Present	200	11.50 [10.40–13.03]	**<0.001**
2M status	Mann–Whitney U	Absent	96	9.39 [8.40–10.72]	**<0.001**
	Mann–Whitney U	Present	218	11.80 [10.80–13.07]	**<0.001**
3M status	Mann–Whitney U	Absent	155	10.30 [8.95–11.70]	**<0.001**
	Mann–Whitney U	Present	159	11.90 [11.10–13.40]	**<0.001**

**Table 6:** Relationship between posterior maxillary morphology, dentition status, and the derived αMT–GPC angular parameter.

Predictor	Test	N/Groups	αMT–GPC Result	*p* Value
PWW-1	Spearman correlation	N = 314	rho = 0.356	**<0.001**
Age	Spearman correlation	N = 290	rho = −0.284	**<0.001**
PPH	Spearman correlation	N = 314	rho = 0.006	0.914
PF–GPC	Spearman correlation	N = 314	rho = 0.208	**<0.001**
1M status	Mann–Whitney U	Absent: n = 114 Present: n = 200	Absent: 34.24 [29.93–38.19] Present: 36.96 [33.33–40.42]	**<0.001**
2M status	Mann–Whitney U	Absent: n = 96 Present: n = 218	Absent: 33.10 [28.62–36.07] Present: 37.16 [33.49–40.36]	**<0.001**
3M present	Mann–Whitney U	Absent: n = 155 Present: n = 159	Absent: 34.92 [30.07–38.80] Present: 37.16 [33.58–39.84]	**<0.001**
PF–GPC threshold-based group	Mann–Whitney U	PF–GPC < 4 mm: n = 69 PF–GPC > 4 mm: n = 245	<4 mm: 34.72 [30.92–38.17] >4 mm: 36.32 [32.17–40.20]	**0.040**

**Notes.** Values for group comparisons are presented as median [IQR] in degrees. MT–GPC is not listed as an independent predictor because αMT–GPC is calculated directly from MT–GPC using αMT–GPC = arctan(MT–GPC/10).

## Data Availability

Data available in a publicly accessible repository 10.6084/m9.figshare.32983139.

## Abbreviations

1M	First maxillary molar
2M	Second maxillary molar
3M	Third maxillary molar
CBCT	Cone-beam computed tomography
CI	Confidence interval
CT	Computed tomography
DICOM	Digital Imaging and Communications in Medicine
FBCT	Fan-beam computed tomography
GPC	Greater palatine canal
HN	Hamular notch
IQR	Interquartile range
MT	Maxillary tuberosity
MT–GPC	Distance between the maxillary tuberosity reference point and the greater palatine canal
OR	Odds ratio
PASA	Pterygoid Anchorage Spatial Adequacy
PF	Pterygomaxillary fissure
PF–GPC	Distance between the pterygomaxillary fissure and the greater palatine canal
PPH	Pterygoid process height
PWW-1	Width of the maxillary alveolar process measured 10 mm anterior to the hamular notch
SCAIS	Static computer-assisted implant surgery
SD	Standard deviation
STL	Stereolithography
